# Behavioral evidence for two-stage sleep in spiders

**DOI:** 10.1073/pnas.2618030123

**Published:** 2026-10-26

**Authors:** Nadja Geiger, Maxim Q. Capelle, Phillip Rosentreter, Marie E. Herberstein, Armin Bahl, Massimo De Agrò, Barrett A. Klein, Daniela C. Rößler

**Affiliations:** ^a^ Section II Animal Diversity, Bonn Institute for Organismic Biology, University of Bonn, Bonn 53121, Germany; ^b^ Department of Biology, University of Konstanz, Konstanz 78464, Germany; ^c^ Zukunftskolleg, University of Konstanz, Konstanz 78457, Germany; ^d^ Centre for Taxonomy and Morphology, Leibniz Institute for the Analysis of Biodiversity Change, Hamburg 20146, Germany; ^e^ Department of Biology, University of Hamburg, Hamburg 22609, Germany; ^f^ School of Natural Sciences, Macquarie University, Sydney, NSW 2109, Australia; ^g^ Center for Mind/Brain Sciences, University of Trento, Rovereto 38068, Italy; ^h^ Department of Biology, University of Wisconsin-La Crosse, La Crosse, WI 54601; ^i^ Department for the Ecology of Animal Societies, Max Planck Institute of Animal Behavior, Konstanz 78467, Germany

**Keywords:** arousal threshold, chronobiology, *Portia*, REM sleep, sleep rebound

## Abstract

Sleep is a fundamental behavioral state found across the animal kingdom, yet most of what we know about sleep is heavily vertebrate-biased. Studies are scarce or nonexistent in all but a few invertebrate lineages. A recent study of an REM sleep-like behavior in a jumping spider has reported unexpected parallels to sleep expression in vertebrates. So far, experimental evidence that these behavioral indicators truly represent sleep was lacking in spiders. Here, we present a comprehensive investigation into sleep behavior in the spider *Portia fimbriata* (Salticidae). We demonstrate diurnal activity, a free-running circadian rhythm, robust sleep architecture, regularly occurring REM-like behaviors, elevated arousal thresholds during those REM-like states, as well as rebound effects following sleep disruption. We conclude that the behavior we observe in spiders is behavioral sleep, providing evidence for an REM-like sleep substate in a terrestrial invertebrate. These results contribute to fundamental questions about the evolution and function of sleep across animals.

## Significance

Sleep is one of the most widespread behaviors in the animal kingdom, yet its origins and functions remain poorly understood because experimental evidence comes almost entirely from vertebrates and a few laboratory model species. We provide a behavioral demonstration for two-stage sleep in a spider, an animal with a considerably different neural architecture and sensory ecology, showing that a jumping spider enters a regulated sleep state with recurring REM-like episodes. By demonstrating sleep in an arachnid lineage using multiple independent criteria, our study expands the taxonomic scope of sleep research.

Recent discoveries in the realm of sleep research—including an REM sleep-like state in spiders (1), two sleep stages in octopuses (2), neural signatures of sleep stages in fruit flies (3, 4), as well as sleep in Cnidaria (5, 6), Platyhelminthes (7, 8), and Nematoda (9)—have emphasized the diversity of invertebrate sleep, and revealed strong parallels to sleep architecture previously only known in mammals and birds. These include periods of inactivity (fruit flies), interspersed with muscular twitches and eye movements (spiders, octopuses), and chromatophore flashing (octopuses), reminiscent of the involuntary movements seen during mammalian REM sleep (10, 11). These discoveries have highlighted the contributions of behavioral observations of sleep, as per its broadly supported definition: 1) characteristic posture, 2) behavioral quiescence, 3) reversibility, and 4) increased arousal threshold (12), with the later added functionally suggestive criterion of 5) homeostatic regulation (13, 14).

Sleep is governed by two interacting regulatory processes: a homeostatic drive that accumulates during wakefulness and dissipates with sleep (Process S), and a circadian pacemaker that aligns sleep timing with daily environmental cycles (Process C) (15–17). In this “two-process model,” homeostatic regulation is expressed as compensatory sleep rebound following deprivation, while circadian control maintains stable rest–activity rhythms synchronized with environmental “Zeitgebers” such as light and temperature (15, 18, 19). Demonstrating that sleep is both actively regulated and rhythmically organized across lineages is crucial for understanding when sleep evolved and how its functions may have diversified in different ecological and sensory contexts (9, 10).

Electrophysiological measures, such as electroencephalograms (EEGs) and local field potentials (LFPs), are still considered the gold standard for establishing sleep and sleep stages in animals (e.g., refs. 20 and 21), however, for most invertebrates, this is simply not (yet) feasible. Animal sleep research has long been stifled by the exclusive focus on mammalian-based criteria, discarding observations that deviated from, or could not be described by these criteria. As a consequence, there is a lack of studies, or even recognition of sleep in invertebrates, resulting in a significant taxonomic knowledge gap. In order to advance our understanding of the origin, functions, and evolution of sleep we must broaden the taxonomic horizon and anticipate similarities as well as lineage-specific differences in the expression of sleep to uncover universal principles (10, 13, 22).

To discover and describe sleep in a broader range of taxa, we and others rely on behavioral criteria (e.g., refs. 10 and 13). By doing so, we can contribute comparative research relating to the functions and evolution of sleep across the animal kingdom. Our focus is sleep in spiders—an unexplored lineage in sleep research.

Spiders offer a promising system for establishing and describing sleep behavior in a noninsect arthropod lineage. They are ancient animals with considerable ecological and sensory diversity (23–25), providing broad comparative potential for linking sleep with sensory ecology and behavior. Even though Tobler’s pioneering early work in scorpions suggested sleep-like states in an arachnid (26), a systematic test of the complete behavioral sleep criteria in spiders (and other chelicerates) is still lacking. The recent report of REM-like behaviors in a jumping spider [*Evarcha arcuata*; (1)] described regularly occurring nocturnal twitching and retinal movements reminiscent of vertebrate REM sleep. Building on Rößler et al. (1) and an earlier report by the same lead author describing rest in a jumping spider (27), we set out to test whether these REM-like episodes occur within a broader, regulated sleep state to provide a full behavioral validation of sleep. Our focal species, the jumping spider *Portia fimbriata*, is a diurnal, visually guided, cognitively complex salticid from tropical Queensland, Australia that can be maintained year-round in the laboratory without diapause. Its clear nocturnal resting posture and visually dominated ecology make it a suitable candidate for rigorous behavioral validation of sleep.

We designed an integrative framework to test the behavioral criteria of sleep in spiders including: 1) Circadian activity tracking to assess whether rest–activity cycles are endogenously regulated (Process C); 2) Quantitative analyses of sleep architecture to characterize the organization of quiescence and REM-like activity, as well as a characterization of the species-specific sleep posture; 3) State-dependent arousal threshold, testing whether responsiveness is reduced during quiescent (and, in spiders, REM-like) states (a key criterion distinguishing sleep from rest); and 4) Sleep disruption experiments to test for homeostatic rebound and thus regulatory control (Process S). Through this integrative framework, we aim to provide a comprehensive behavioral validation of sleep in a spider and to establish spiders as comparative models for exploring the evolution and function of sleep.

## Results

To characterize circadian rhythms and sleep in *P. fimbriata*, we used several complementary behavioral approaches, including locomotor activity tracking, detailed behavioral scoring of sleep architecture, arousal threshold tests, and sleep disruption protocols. All experiments were performed under controlled environmental conditions. Throughout this study, we use “sleep window” as an operational term encompassing sustained periods of behavioral quiescence that entail sleep, bouts of REM-like sleep, as well as quiet wakefulness. The onset of the sleep window is defined as the adoption of a resting posture combined with the cessation of movement. We then marked the end of the sleep window with the start of movement at the end of the night (for further details, see Table 1).

**Table 1.** Behavioral definitions

Event	Event type	Description
(1) Sleep-associated events		
Sleep onset	Point	Moment the spider first adopts a sleep posture and becomes quiescent.
Sleep offset	Point	Moment quiescence terminates at the end of the sleep phase, indicated by the spider initiating slow, coordinated movements.
Sleep bout	State	Prolonged inactivity with characteristic drooping posture; includes REM-like bouts and quiet wakefulness if applicable.
Sleep positioning	State	Settling into the characteristic sleep posture prior to a sleep phase.
REM-like bout	State	Twitching/movements of opisthosoma, spinnerets, chelicerae, pedipalps, or legs, and random retinal-tube movements when visible; occurring within sleep bouts.
Leg curl	State	Curling of one or more legs, typically marking the onset or continuation of a REM-like state.
Nap	State	Short sleep episode occurring between active periods during the day.
(2) Wake-associated events		
Wake (night)	State	Brief episode of wakefulness within the typical sleep phase.
Movement (day)	State	General locomotor activity during the active period.
Inactivity (day)	State	Stationary posture with minor movements or short pauses within active period.
Grooming	State	Grooming of legs or pedipalps in a controlled sequence.
(3) Temporal and experimental markers		
Lights off	Point	Onset of the subjective dark phase (night).
Lights on	Point	Onset of the subjective light phase (day).
Baseline night	State	Night preceding the sleep-disruption manipulation.
Baseline day	State	Day preceding the sleep-disruption manipulation.
Rebound night	State	Night following sleep disruption.
Rebound day	State	Day following sleep disruption.

Observations were annotated as either state events, representing behaviors of measurable duration, or point events, denoting brief, instantaneous actions. Although sleep onset is likely a gradual process, it was scored as a point event to enable consistent temporal alignment. Temporal markers (e.g. lights on/off, baseline vs. rebound phases) were scored in BORIS alongside behavioral events to enable precise data alignment and phase segmentation.

We first analyzed sleep–wake rhythms by using lack-of-motion and locomotory activity as proxies for sleep and wake, respectively. We then analyzed sleep architecture at high temporal resolution, incorporating posture, immobility, and REM-like behaviors to identify the states most likely to represent sleep. Finally, arousal threshold measurements and sleep disruption experiments were used to test if REM-like states are associated with reduced vigilance and to demonstrate homeostatic regulation, providing functional validation that the observed quiescent states constitute true sleep.

### Endogenous Circadian Control of Activity.

**Fig. 1.**
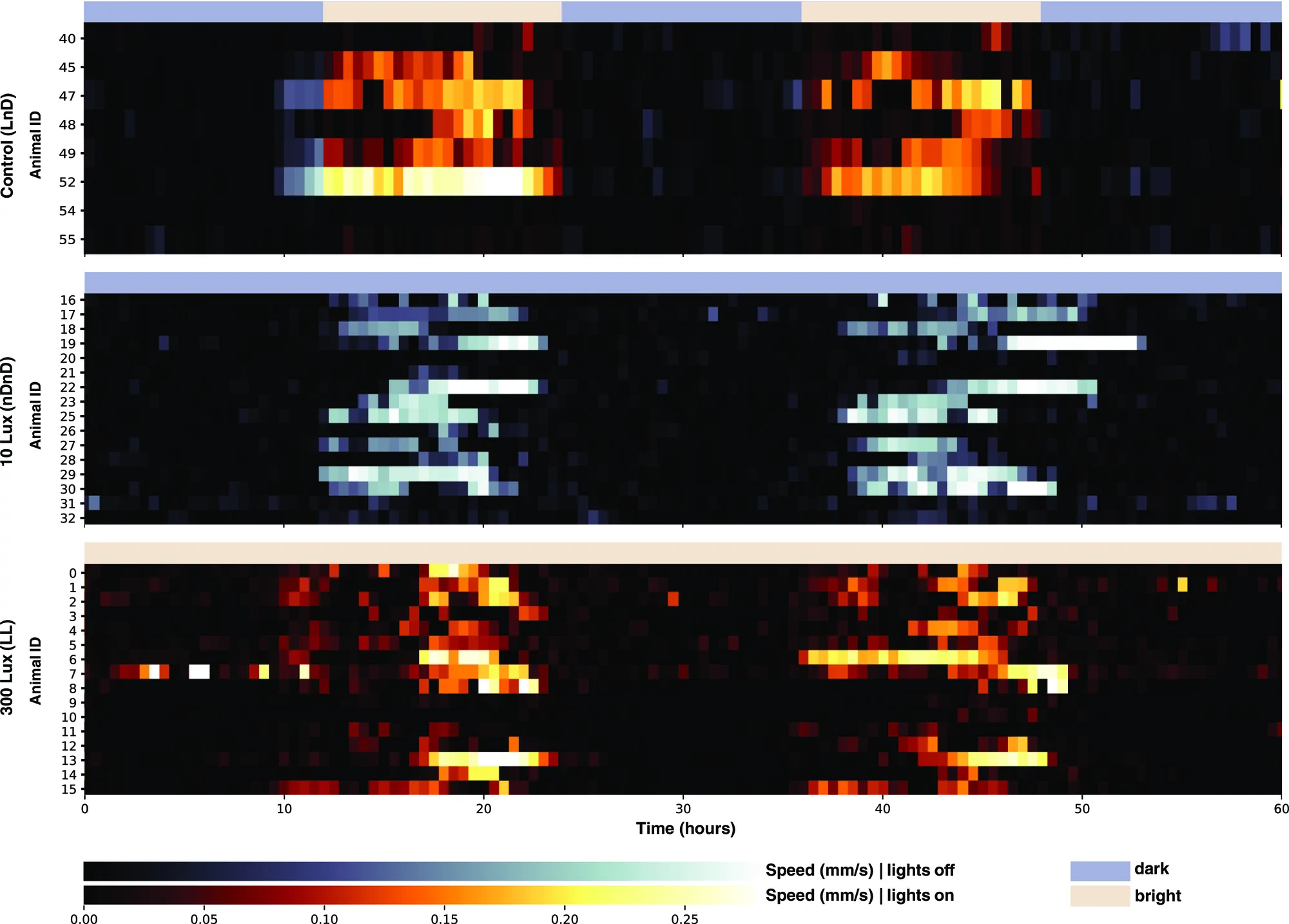
Heatmaps of spider activity across 60 h. The x-axis represents time (hours) after first “lights off” (0 h) except for the LL treatment where lights stay on throughout the 60 h. The light–dark cycle was completely aligned with the LD cycle in the laboratory with 0 h marking the normal lights off time. The y-axis indicates individual spider identity. Color intensity represents mean locomotor speed (mm/s), binned at 5 × 10^−4^ Hz (30-min intervals). The *Upper*, *Middle*, and *Lower* panels correspond to the LnD, nDnD, and LL groups, respectively. Periods of near-darkness are depicted using a black-blue-white color scale, whereas periods of light are represented using a black-orange-white color scale. Additionally blue and beige bars on top of each heatmap visualize “lights on” and lights off periods.

Activity patterns across different light treatments over a consecutive recording period (60 h) are summarized as heatmaps in Fig. 1. Under a 12:12-light–near-dark (LnD) treatment, most spiders showed a clear daily pattern, with activity predominantly concentrated during the light phase, and prolonged periods of inactivity during the near-dark phase.

A similar alternation between periods of higher and lower activity remained visible under constant near-darkness (nDnD), despite the absence of an external light–near-dark transition. Inactivity remained comparatively consolidated, and successive periods of increased activity occurred at daily intervals across the recording period.

Under constant light (LL), activity was initially more irregular, particularly in the first circadian cycle (see spider ID 7). Thereafter, an alternating pattern of higher and lower activity became apparent again. However, compared with LnD and nDnD, periods of low activity were less consolidated and activity was more fragmented across the circadian cycle under LL conditions.

Across all treatments, individuals varied in their overall activity levels. Some spiders showed longer periods with little or no detectable movement, whereas others were consistently more active (i.e., exhibited movement at greater speed). Despite this interindividual variation, the overall temporal patterns of lower and higher activity periods remained apparent across treatments.

### Sleep Architecture: Consolidated Sleep and REM-Like States in *P. fimbriata*.

**Fig. 2.**
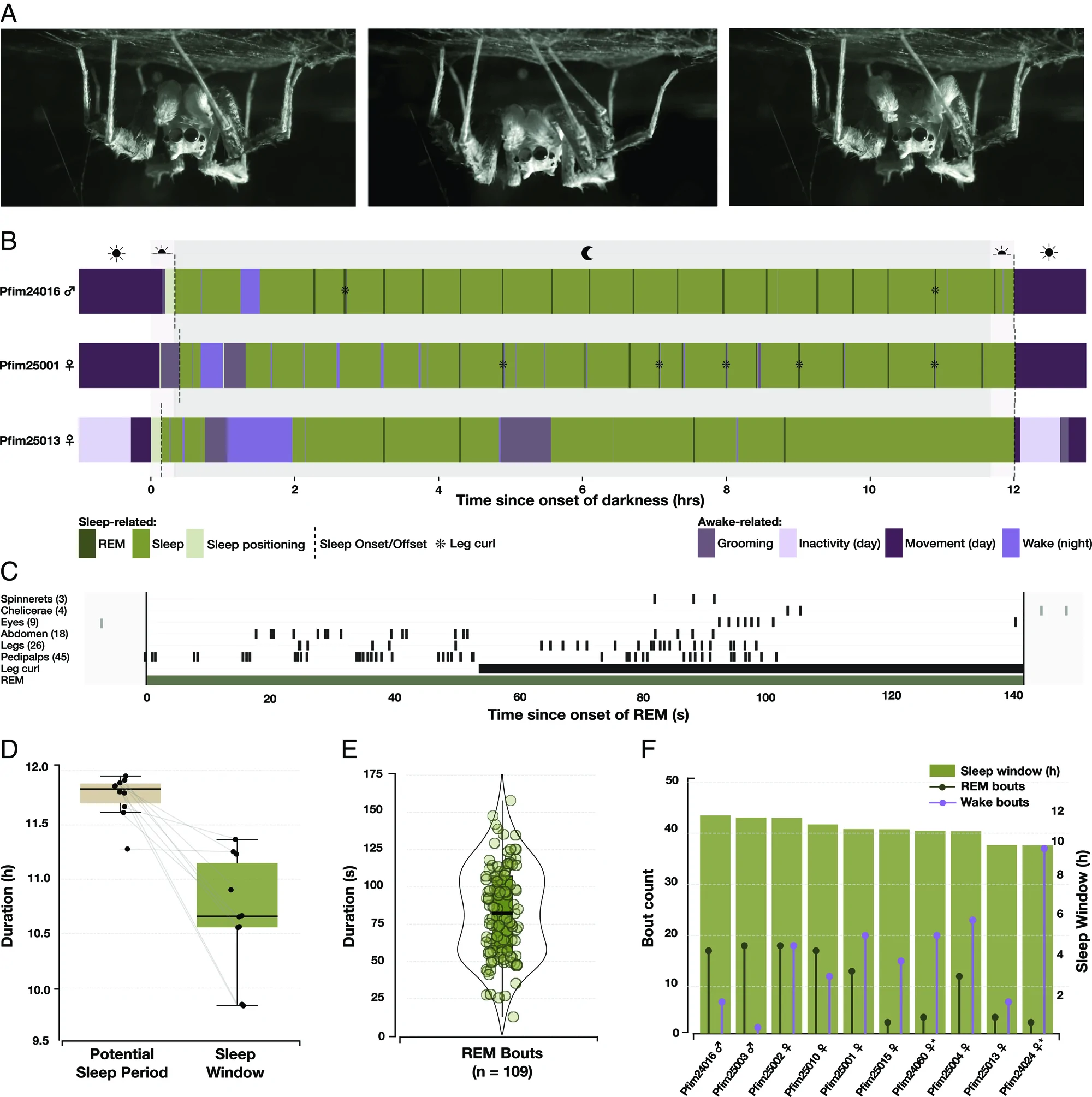
Sleep posture and architecture in *P. fimbriata*. (*A*) Posture comparison. (*Left*) Sleep initiation: upside down position under web, pedipalps drawn in. (*Middle*) Sleep: body and pedipalps visibly drooped, hanging lower under the web. (*Right*) Waking up position: pedipalps spread, body tenser, closer to web. (*B*) Ethogram showing temporal organization of behavioral states and events in three *Portia* spiders visualizing variation as well as consistency in sleep architecture. Distribution of distinct behavioral states (sleep, REM-like, leg curls, wake, grooming, positioning) over a 14-h period starting 1 h before and ending 13 h after the onset of darkness. Each spider is represented in its own horizontal row. Vertical dashed lines indicate sleep on- and offset; light transitions (lights-off at 0 h, lights-on at 12 h) are marked by background shading (dark phase = dark gray; twilight = light gray; light phase = no shading). Ethograms for all 10 individuals can be found in the supplemental material (*SI Appendix*, Fig. S1). (*C*) Ethogram demonstrating an exemplary REM-like bout with associated behavioral markers. REM-like state and leg curl are shown as horizontal bars. Short bars above indicate point events (twitching of pedipalps or other legs, abdomen, spinnerets, and chelicerae, or eye movements, respectively; numbers in brackets indicate number of occurrences). Time is shown relative to REM-like onset (0 s), with the gray area indicating a 10-s pre-REM-like period. (*D*) Boxplots comparing the duration of Potential Sleep Period (mean ± SD: 11.8 ± 0.2 h) and Sleep Window (h); mean ± SD: 10.7 ± 0.5 h). (*E*) Violin plot; pooled duration of REM-like bouts. (*F*) Barplots [duration of sleep window (h)] with pairwise comparisons of REM-like and wake bouts per spider.

Our behavioral analyses from continuous infrared video recordings found evidence of diurnal activity in *Portia*. Throughout the day, *Portia* displayed a distinct behavioral profile compared to nighttime. While they frequently remained stationary for extended periods, they were rarely truly inactive. Even in apparent stillness, they actively monitored their surroundings—turning, scanning, or adjusting their posture in response to visual stimuli. They also engaged in locomotion, web-building, or grooming, maintaining a state of alertness, and engagement typical of their complex predatory lifestyle. This low intensity yet continuous engagement in purposeful movement contrasted with their behavior during the night. The transition into sleep was marked by a gradual withdrawal from these activities. As night approached, spiders began searching for their sleeping site, often a silk retreat, and engaged in prolonged grooming. Once settled, they adopted a stereotypical sleeping posture. Unlike the suspended resting posture in *E. arcuata* (1, 27), *Portia* sat motionless on a horizontal silk platform with the dorsal prosoma facing downward, and the opisthosoma (major front and hind segments of body, respectively) starting to droop gradually—a subtle change that became apparent when reviewing sped up video recordings (see also Movie S1). During this position, the pedipalps were drawn inward and held close together beneath the prosoma, differing from the open, more laterally positioned pedipalps during wakefulness (Fig. 2*A*). This posture was maintained for the majority of the night. All individuals exhibited nocturnal quiescence characterized by distinct periods of behavioral inactivity (see also Fig. 2*B* and *SI Appendix*, Fig. S1). Spiders maintained a consistent sleep window (sleep onset to offset) that closely aligned with the dark phase of the 12:12-h light–dark cycle (Fig. 2*D*), with sleep onset typically occurring within the first 30 min of darkness (14.0 ± 12.4 min), and waking at the onset of light (−0.3 ± 2.7 min). The sleep window was largely consolidated, with only brief interruptions (1.3 ± 0.8 wake bouts per hour) due to repositioning or grooming behaviors (13.2 ± 8 wake bouts per night, with mean wake bout durations of 324.7 ± 322.3 s; grooming events (8 ± 5 per night); Fig. 2*F*). Despite occasional fragmentation, the sleep window lasted the majority of the night (mean ± SD: 10.7 ± 0.5 h, see Fig. 2*D*).

Spiders cycled between extended periods of behavioral quiescence and short bouts marked by rapid retinal movements, twitching, and leg curling—the same behaviors that were previously reported as REM sleep-like states in the salticid *E. arcuata* (1); see also Figs. 2 *B* and *C* and 5 and Movie S1). Across all individuals (n = 10), we recorded 109 REM-like bouts, with an average of 10.9 ± 6.7 REM-like bouts per night and spider, each lasting 82.6 ± 14.7 s (Fig. 2*E*). The first REM-like episode occurred 240.0 ± 184.7 min (min–max range: 67.5 to 600.7 min) after sleep onset, highlighting substantial interindividual variation in REM-like timing. Approximately 38.8% of bouts (unweighted) included leg curling, consistent with the proportion reported for *E. arcuata* [39.5%; (1)].

### Arousal Threshold: State-Dependent Differences.

**Fig. 3.**
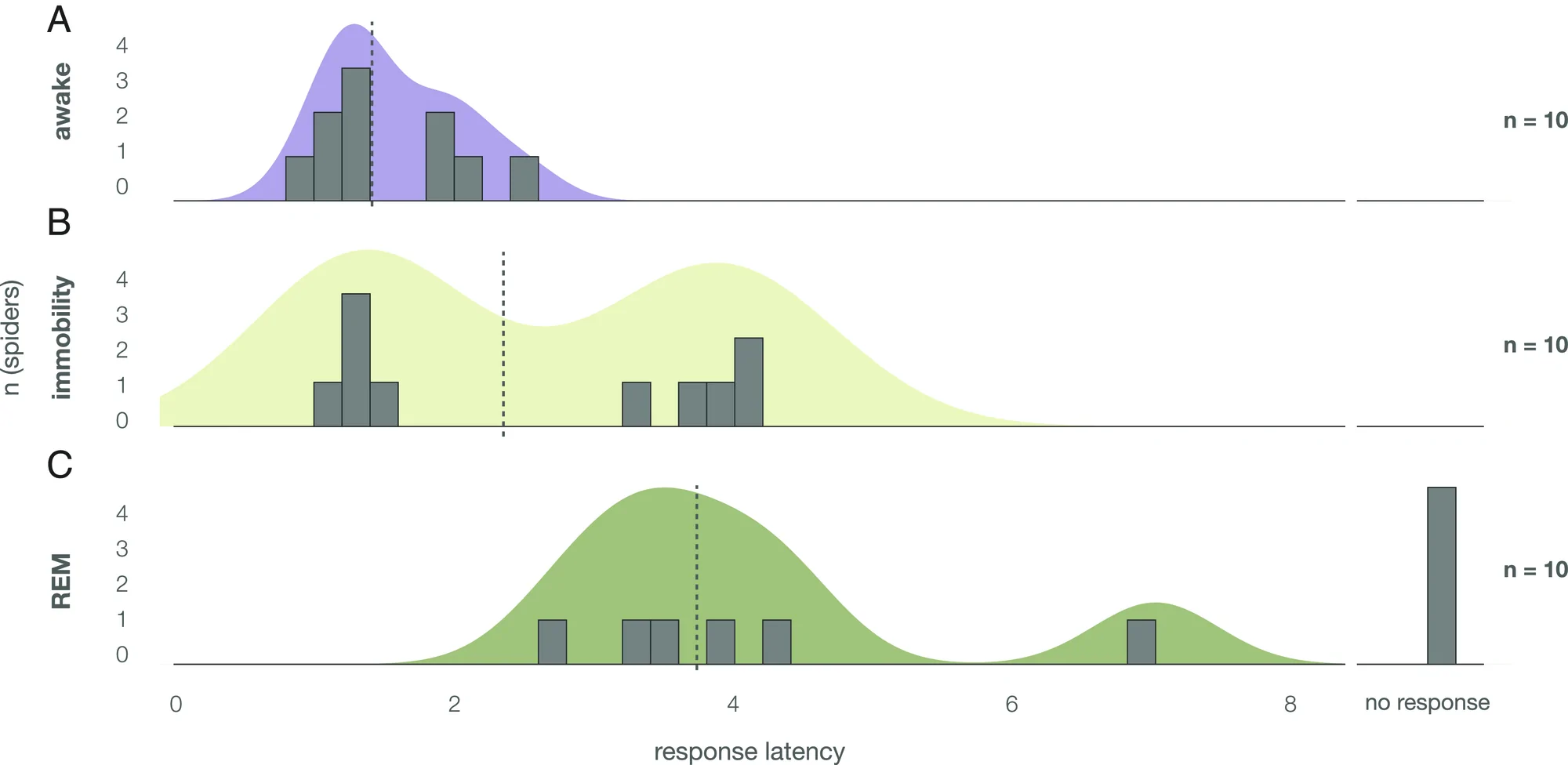
State-dependent differences in response latency in *P. fimbriata.* Response latency in seconds to sound stimulus of *P. fimbriata* in different states: (*A*) Awake, (*B*) Immobility (>15 min immobile), and (*C*) REM-like.

Behavioral state significantly affected reaction latency to a standardized auditory stimulus (GLMM, χ² = 23.9, df = 2, *P* < 0.0001, n = 26 responding trials, see Fig. 3), whereas sex did not (χ² = 0.06, df = 1, *P* = 0.81). Among responding spiders, latencies were shortest during wakefulness (estimated mean ± SE: 1.69 ± 0.20 s), intermediate during quiescence (2.71 ± 0.32 s), and longest during REM-like episodes (4.27 ± 0.65 s) (see also Movie S2). Post hoc comparisons showed that reaction latencies were significantly longer during quiescence than during wakefulness (awake vs. quiescence, *P* = 0.0137) and significantly longer during REM-like episodes than during wakefulness (awake vs. REM, *P* < 0.0001), whereas REM-like episodes showed a trend toward longer latencies than quiescence, although this difference did not reach statistical significance after Bonferroni correction (*P* = 0.0548). Descriptively, responsiveness was also reduced during REM-like bouts: all spiders responded within the 10-s testing window when awake and during quiescence (10/10 each), whereas only 6/10 responded during REM-like episodes and four spiders failed to respond entirely. These initially unresponsive individuals responded immediately when retested while awake, confirming state-dependent suppression of responsiveness rather than sensory or motor impairment. Responses during quiescence were intermediate overall and appeared heterogeneous: some spiders responded as rapidly as during wakefulness, whereas others showed latencies comparable to those observed during REM-like episodes.

### Sleep Disruption: Evidence for Homeostatic Rebound.

**Fig. 4.**
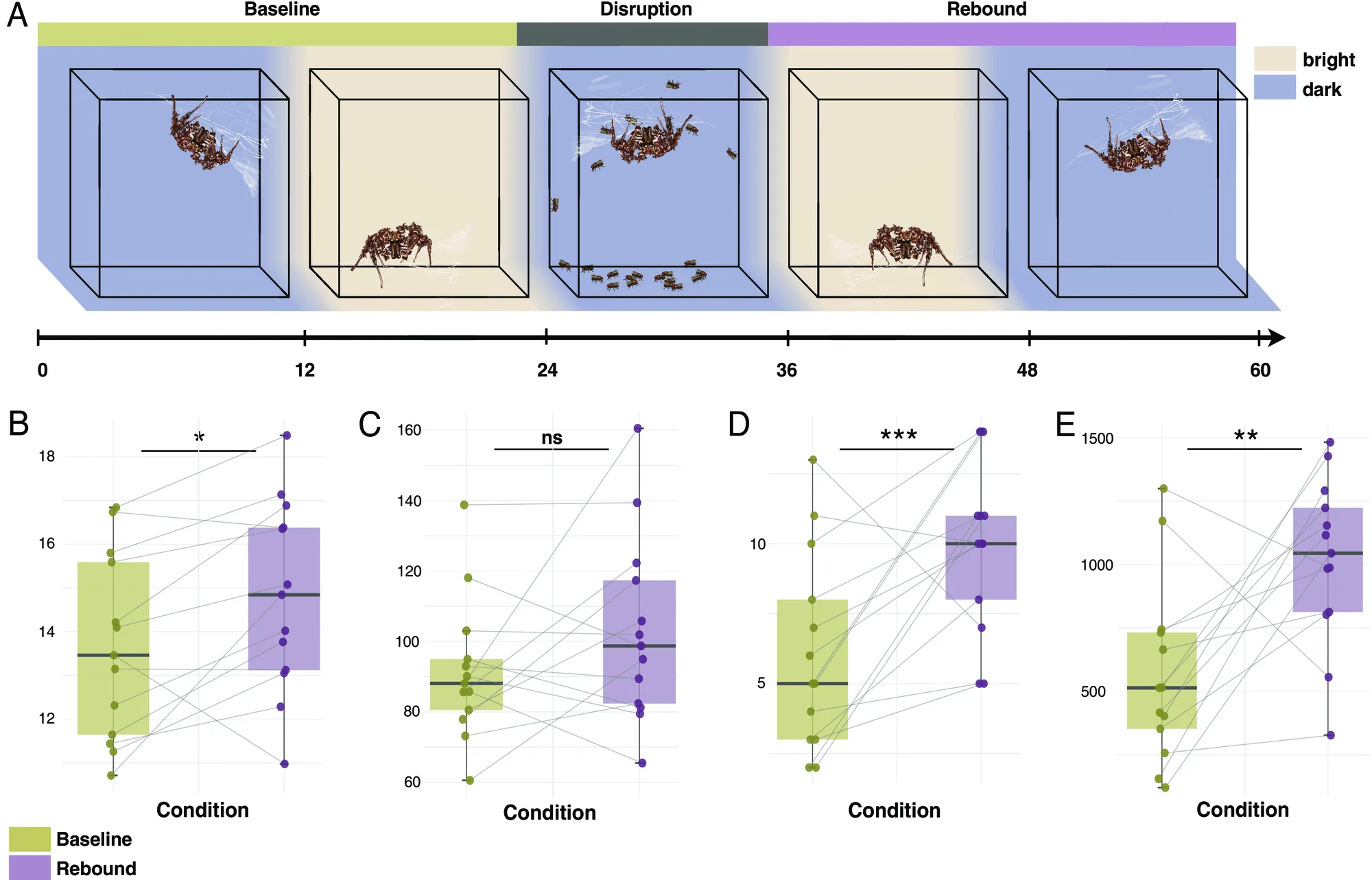
Setup and effects of sleep disruption. Conditions: Baseline vs. Rebound phases. (*A*) Setup schematic and experimental timeline. The schematic illustrates the experimental phases (top bar) across time (h; x-axis). Dark phases are shown in blue and light phases in yellow. Boxes represent filming enclosures. Spider illustrations represent *Portia* in characteristic sleep or awake postures, and fly illustrations denote the presence of the *D. melanogaster* stimulus during the disruption phase. (*B*) Total sleep window (h) by paired condition. (*C*) REM-like bout duration (s) by paired condition. (*D*) Number of REM-like bouts by paired condition. (*E*) Total time spent (s) in REM-like state by paired condition. Significance levels: ns, not significant; *P < 0.05; **P < 0.01; ***P < 0.001.

To test for homeostatic regulation, spiders were exposed to one night of sleep disruption via continuous disturbance with *Drosophila melanogaster*. We then quantified behavioral variables during baseline and rebound phases, focusing on total sleep window, REM-like activity, and REM-like bout structure. Spiders showed compensatory changes following sleep disruption. The total sleep window increased significantly during the rebound night relative to baseline (mean ± SE: 13.64 ± 0.59 h vs. 14.80 ± 0.60 h; +8.5%; GLMM, χ² = 6.32, *P* = 0.012, n = 13; Fig. 4*B*). Although the duration of individual REM-like bouts remained unchanged (baseline: 93.0 ± 4.4 s, n = 79 bouts; rebound: 101.6 ± 4.2 s, n = 130 bouts; GLMM, χ² = 1.62, *P* = 0.20; see Fig. 4*C*), REM-like bouts occurred significantly more often during rebound (6.1 ± 1.0 vs. 10.0 ± 0.9 bouts; +64.6 %; GLMM, χ² = 12.19, *P* < 0.001; Fig. 4*D*). Consequently, total REM-like time also increased significantly from baseline to rebound (9.4 ± 1.7 min vs. 16.9 ± 1.5 min; +79.8%; GLMM, χ² = 8.60, *P* = 0.0034; Fig. 4*E*). Furthermore, when expressed relative to the sleep window, REM-like time increased from 1.18 ± 0.77% (baseline) to 1.95 ± 0.68% (rebound), representing a +65.1% relative increase. In the undisturbed control experiment, sleep window duration did not increase across three consecutive nights, averaging 12.0 h on nights 1 and 2 and 11.6 h on night 3. Night significantly affected sleep window duration (GLMM, χ² = 14.54, *P* = <0.001, n = 5), with no difference between night 1 and 2 (*P* = 1.00), but a shorter sleep window on night 3 compared to night 1 (*P* = 0.0019) and 2 (*P* = 0.0039; *SI Appendix*, Fig. S2).

## Discussion

Sleep has long been defined and understood through the lens of vertebrate neuroscience, which leaves open the question of whether, and in what form, sleep is expressed in animals with considerably different nervous systems and sensory worlds. Here, we show that the jumping spider *P. fimbriata* enters a sleep state that is homeostatically regulated, structured, and possibly also functionally differentiated (multiple sleep stages). Based on multiple independent experiments, we show that *Portia* met all established behavioral criteria for sleep, including a characteristic posture, consolidated quiescence, circadian organization, elevated arousal thresholds, and homeostatic rebound after disruption. Taken together, these findings provide comprehensive behavioral evidence for sleep in a spider.

Sleep in *Portia* is embedded within a circadian framework. Activity rhythms persisted under constant near-darkness and became weaker, although they did not disappear under constant light. This is consistent with endogenous circadian control of rest–activity cycles. The persistence of rhythmic organization in the absence of external Zeitgebers is considered evidence for an internal clock regulation. Our data support the finding that circadian timing mechanisms are conserved across the animal kingdom, including invertebrates (e.g., insects and arachnids; 19, 28–30).

Establishing the circadian phase and rhythm stability was important for interpreting when sleep occurred and how it was structured in *Portia*. In many species, the circadian organization provides the temporal framework within which sleep and REM-like activity are arranged [but see exceptions, such as cathemeral sleepers (31)]. For a visually guided predator such as *Portia*, living in complex, three-dimensional environments, concentrating sleep at night may reflect a balance between sensory constraints and ecological pressures. Vision is less reliable under low light and may be combined with greater predation risk from nocturnal predators. Under those conditions, behavioral withdrawal during the night may be favored, while visual acuity and cognitive performance are preserved for daytime foraging. Continuous high-resolution infrared video tracking also allowed us to quantify locomotor rhythms without relying on coarser activity proxies such as beam crossings (e.g., ref. 32). High resolution tracking captures fine-scale behavioral dynamics that are often missed by traditional circadian monitoring systems, and may be especially useful for studying sleep in small, or nonmodel species.

Within this circadian framework, the sleep window in *Portia* does not appear to be a uniform state but shows an internal architecture. Long bouts of behavioral quiescence are interrupted by distinct REM-like episodes marked by twitching, retinal movements, and leg curls—the very same behaviors recently described in the jumping spider *E. arcuata*, with similarities to vertebrate REM sleep behavior (1). The recurrence and modulation of these phases suggest the presence of distinct sleep states that may have different functions. The expression of REM-like activity in *Portia* can be interpreted in light of the species’ sensory ecology and cognitive abilities. Living in the dense, stimulus-rich rainforest understory, *Portia* may have to process a great amount of sensory information (e.g., motion, contrast, spatial structure, silk, and prey cues) across multiple dimensions and light conditions (33, 34). Its sophisticated visual system, together with its abilities related to detour planning, object recognition, flexible problem-solving and decision-making behaviors (33, 35, 36) likely impose substantial demands on neural processing and memory. In that context, periodic REM-like activation during sleep may indicate a brain state that supports synaptic plasticity, sensory recalibration, or the integration of experience, analogous to work, particularly in mammals, linking REM sleep to neural reorganization and learning (e.g., refs. 37–41). Taken together, this makes *Portia* a promising system for associating sleep with cognition and predation. As a predator that relies on flexible decision-making, planning, and visually guided attack strategies, *Portia* offers opportunities to explore how sleep interacts with behaviors that are directly relevant to survival and fitness. Further exploration of the at least superficial resemblance of *Portia* REM-like sleep to mammalian REM sleep could involve manipulating spider orthologs of Chrm1 and Chrm3 genes, or acetylcholine neurotransmitters known to regulate mammalian REM (see refs. 42 and 43).

Our data indicate that sleep in *Portia* is further distinguishable from passive inactivity because it is state dependent. We found that the responsiveness to auditory stimulation was reduced during both quiescent and REM-like states compared with wakefulness, and some individuals did not respond at all during REM-like states. State-dependent unresponsiveness is a core feature of sleep across lineages, and supports the interpretation that REM-like episodes represent a distinct sleep state, consistent with patterns observed in both invertebrates and vertebrates (2, 9, 21, 44–46). Response latencies during quiescence showed variability, suggesting that immobility in *Portia* includes both lighter, more alert rest, and deeper, sleep-like states. This would parallel patterns reported for *Araneus diadematus* (47), and is consistent with the view that sleep exists along a continuum of states rather than a binary switch between sleep and wakefulness. However, given the relatively small sample size, this interpretation remains tentative and requires further testing. Despite the state-dependent differences in responsiveness observed here, without neurophysiological information, we cannot exclude the possibility that portions of the sleep window represent quiet wakefulness without a behaviorally observable state change, as in other studies identifying sleep primarily from behavioral observation (e.g., refs. 48–50). While sleep is often scored using a classical inactivity threshold [i.e., × minutes of inactivity; e.g., in elephants: (51)], our approach also incorporates a species-specific sleep posture when defining sleep onset. Future experiments should aim to disentangle potentially distinct states within these periods of quiescence using more arousal threshold testing, ideally complemented by neurophysiological recordings.

Following sleep disruption, our data support a homeostatic rebound, with longer sleep window duration and a near doubling of total REM-like time, without alterations in bout spacing or duration. The observed rebound after sleep disruption cannot be explained by a general tendency for sleep window duration to increase across successive nights, as control experiments showed. The fact that this temporal structure was preserved, despite increased recovery need, suggests that sleep architecture is stably maintained while the number of REM-like events can be adjusted more flexibly to rebound. Together, these findings support the idea that *Portia’s* sleep is an actively regulated physiological process, balancing sleep pressure and restoration—features cited as evidence for true sleep across animals (13, 14). *Portia* REM-like episodes regularly occurred within a consolidated sleep window, were associated with the strongest reductions in responsiveness, and were regulated following sleep disruption. Taken together, these patterns provide the strongest evidence to date for an REM-like sleep substate in a terrestrial invertebrate.

Although consistent patterns emerged throughout all experiments, individuals still differed in sleep timing, architecture, responsiveness, and REM-like expression. This variation may point to a degree of plasticity that allows sleep to be adjusted to internal state and environmental context. It also raises possibilities for future work on developmental effects (see ref. 52), reproductive state, and experience-dependent change.

We hope our work will contribute to the development of conceptual and methodological frameworks as envisaged by Rößler and Klein (21). By embracing the classical and broadly accepted behavioral framework, it becomes possible to identify and compare sleep across a broader range of taxa, enabling comparative sleep research that spans the full diversity of animal life.

Sleep is among the most conserved yet most variable behaviors in the animal kingdom, and its origins may become clearer not only through similarities among established models, but through the differences expressed across lineages (10, 22). With their ancient evolutionary history, diverse ecologies, and rich sensory worlds, spiders are well positioned to test how evolutionary pressures shape sleep architecture and function. Questions that emerge from our work include examining how sleep varies across spiders with different sensory priorities, how or whether sleep changes developmentally as well as under natural disturbance and predation risk, and how sleep quality feeds back into waking cognition and performance.

## Materials and Methods

### Spider Husbandry.

Juvenile *P. fimbriata* were housed individually from the second to third instar onward in clear plastic containers (6 × 6 × 15.5 cm) and transferred to larger containers (10 × 10 × 18 cm) prior to the fifth instar. Each container was equipped with a 2-mL water-filled Eppendorf tube stoppered with cotton for hydration, plus wood wool, leaf debris, and sticks to facilitate web-building and resting. Conditions matched their native Queensland (Australia) habitat: 12:12 h light–dark with 30-min dawn and dusk simulated by stepwise 15-min brightness transitions; daytime temperature 26 ± 2°C, with ~3°C drop at night; ~60% relative humidity. Spiders were fed twice weekly with a varied diet of other spiders, flies, and crickets, following published recommendations (53).

### Infrared Video Recordings.

Unless stated otherwise, recordings (30 fps) used infrared-modified DSLR cameras (Nikon D7200 and Nikon D7500) equipped with AF-S Micro NIKKOR 60 mm, f/2.8G ED lenses; +10 diopter close-up lens (Walimex, 62 mm), were added for small juveniles. External 940 nm LED panels (VBESTLIFE 140 LED) provided illumination without affecting spider behavior.

We combined three complementary recording approaches to maximize data accumulation and filming capacity. We chose the most practical option, or a combination, for each setup (described in the relevant sections below): 1) DSLR time-lapse capture: programmable intervalometers (Rollei cable remote control 28005) triggered successive 30-min segments to bypass DSLR recording limits. This introduced ~10 s gaps between clips, which were marked and excluded during postprocessing but had negligible impact on behavioral scoring. 2) HDMI streaming to computer: continuous live video was streamed using capture cards and recorded with OBS Studio (Open Broadcaster Software, V.32.0.1), producing uninterrupted recording sessions. 3) HDMI streaming to external recorders: direct recording via HDMI recorders (AVerMedia EzRecorder) to SSDs (4TB SanDisk), enabling gap-free, high-capacity capture without a connected computer. If spiders molted or left the frame, the individual was recorded on a subsequent date.

### Environmental Conditions.

All experiments were conducted at 26°C ± 2°C during the day, with a ~3°C drop at night, and at ~60% relative humidity under a 12 h:12 h light–dark cycle with twilight transitions as described above.

### Ethics Statement and Permits.

This study was conducted on invertebrates for which formal ethics approval is currently not required. We followed the guidelines for the treatment of animals in behavioral research by the Association for the Study of Animal Behaviour [ASAB, (54)]. All spiders (n = 92) were *P. fimbriata* (hereafter *Portia*) offspring of animals sourced from a captive breeding program in Queensland, Australia (minibeast; export permit number PWS2023-AU001805).

### Experimental Protocols.

#### Circadian activity tracking.

To assess locomotor activity and endogenous rhythmicity, we tracked spiders individually (n = 40) under different light conditions using a high-throughput infrared-based behavioral tracking system (as described in ref. 55). Spiders were tested in circular arenas (12 cm diameter, 2 cm height) under three light conditions: a 12:12 h light–near-dark (LnD) baseline (300 lx day/10 lx night; n = 8), constant near-darkness (nDnD; 10 lx; n = 16), and constant light (LL; 300 lx; n = 16). Spiders tested in the baseline LnD were introduced to the arenas about 3.5 h before the trial start (lights off), in the nDnD and LL condition about 7 h before the trial start. The light–dark cycle before the experimental period was 12:12 (*Spider Husbandry*) and completely aligned with the cycle of the setup: zero (trial start) marked the time when lights would normally switch off in the laboratory. The nDnD treatment resulted in a near-darkness illumination, due to marginal residual light produced by the projector (angled away from the animal). Trials lasted 60 h; the LnD condition had a smaller sample size due to equipment availability and occasional recording interruptions. The arenas were housed in fully light-controlled enclosures with infrared illumination from below (940 nm) and monitored using infrared-sensitive cameras (Basler acA2040-90um-NIR), equipped with an adjustable macro lens (Navitar ZOOM 7000) and an IR longpass filter (Linghuang Zomei IR 850 nm 52 mm). Light conditions were projected from below using an AAXA P300 Pico Projector. To ensure control over the projected brightness levels, we measured intensities for a fixed interval of projector input values using an LX1330B light meter (Hongkong Thousandshores Ltd., Central Hong Kong). Subsequently, a sigmoid function was fitted to the measured values for each projector and inverted to retrieve the appropriate projector input value for each desired brightness level.

Spiders were fed 1 d before trials but not during recordings, and hydration was provided via a moist cotton ball within each arena.

Locomotor activity was extracted from 30-Hz tracking data, downsampled to 1-Hz, and analyzed based on average movement (speed; mm/s). Animal position was detected by background-subtraction tracking, in custom Python software (see ref. 56). Data from one individual (subject 52, hours 21 to 23) were excluded due to tracking errors probably caused by lighting and camera malfunction.

For each subject, missing speed values from individual frames were first estimated by linear interpolation. To characterize general activity patterns rather than isolated locomotor events, speed data of all spiders were subsequently binned at a frequency of 5 × 10^−4^ Hz, corresponding to 30-min intervals. Finally, three separate heatmaps were generated, one for each experimental group (LnD, nDnD, LL), with the activity of each individual subject displayed in a separate row (Fig. 1).

#### Sleep architecture recording.

**Fig. 5.**
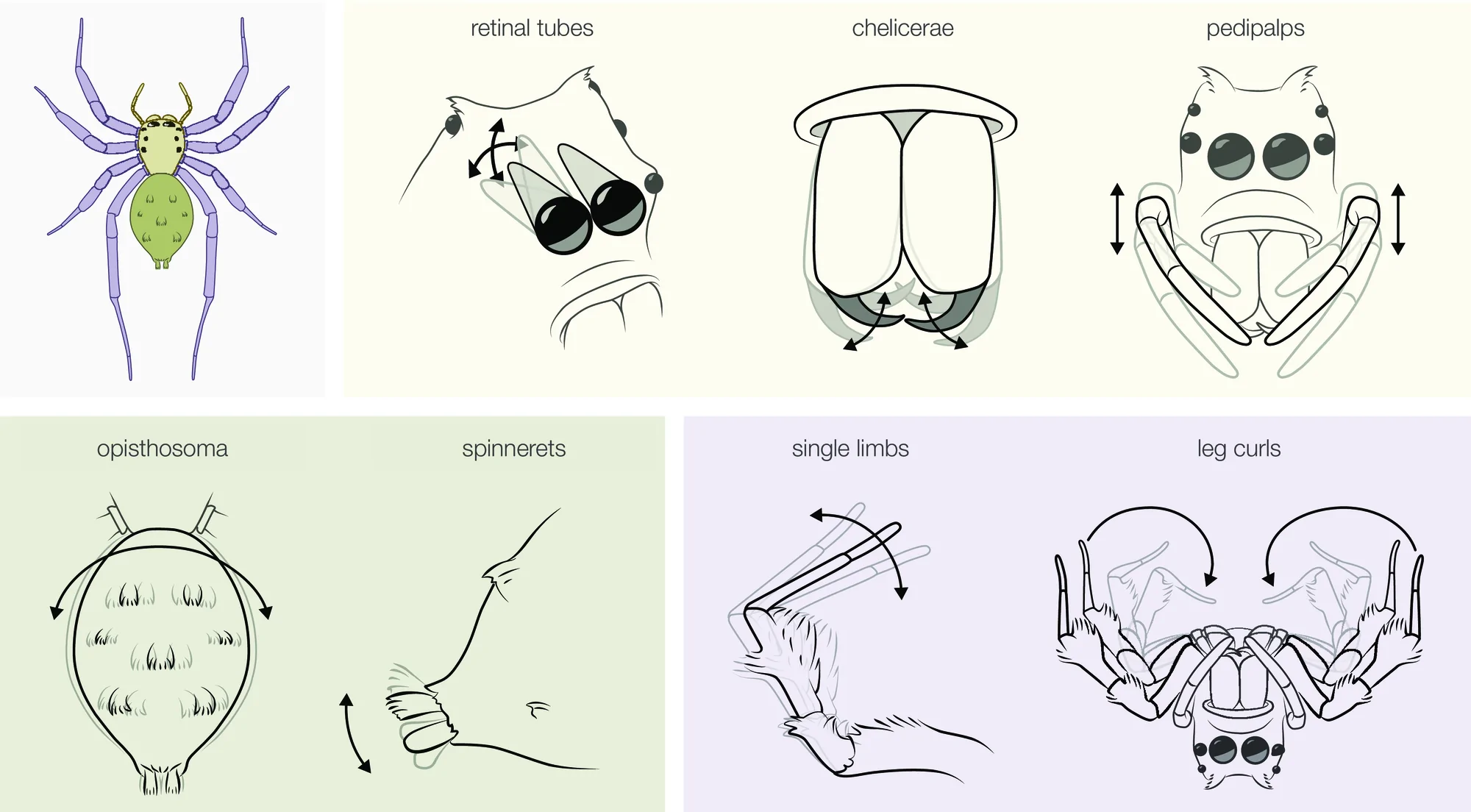
Behavioral markers of REM-like sleep in *P. fimbriata*. Illustration by Maartje van Denderen.

To investigate the structure of rest and activity in *Portia*, we analyzed infrared video recordings from 10 individuals: 8 subadult (2 males, 6 females) and 2 adult spiders (both females) from the baseline nights of the sleep homeostasis experiment (see below). Spiders were recorded continuously for 14 h (12 h night plus 1 h before and after the night period) in individual transparent filming enclosures under standard light and environmental conditions described above. Behavioral states were manually annotated using a predefined ethogram (see below, Table 1). Sleep-associated states comprised behavioral quiescence, characteristic sleep positioning, and REM-like episodes (twitching/movements of different body parts and leg curling; see Fig. 5). Leg curling refers to a passive flexion of the legs that likely reflects a transient reduction in muscle tone and hemolymph pressure, consistent with the hydraulic mechanism of leg extension in spiders (57) and reminiscent of muscle atonia observed during REM sleep in vertebrates (46). Awake-associated states included, e.g., locomotion, grooming, and repositioning (Table 1 and Fig. 2*B*). We hereby differentiated between “movement (day),” which entailed actively moving around during the daytime period, and “wake (night),” meaning brief waking movements within the consolidated sleep window, which never entailed covering distance. “Inactivity (day)” described extended periods of immobility during the day before moving to the sleeping site and adopting a resting position.

#### Arousal threshold.

We assessed arousal thresholds in adult *Portia* (n = 30; 15 males, 15 females) by presenting a standardized auditory stimulus during three distinct behavioral states: 1) REM-like activity, 2) behavioral quiescence, and 3) active wakefulness. Each spider was tested once and in a single behavioral state (10 individuals per state). Spiders were acclimated ≥2 h in transparent vials (3-cm diameter, length = 8 cm; no lid) to allow settling and the web construction at the sleep site (“hammock”). Vials were mounted upside down on a custom 3D-printed platform. A mini Bluetooth speaker (Eageroo) was embedded beneath the platform through a cut-out, and delivered a 400-Hz sine wave, increasing linearly in amplitude over 10 s (generated via Audio Function Generator app, iPad system volume 50). Vials were illuminated laterally with infrared light to allow live observation and video recording.

The behavioral state was confirmed before stimulation: REM-like activity (retinal movements, twitching, leg curling), quiescence, or active wakefulness (grooming or similar activities). Quiescence was defined as uninterrupted immobility lasting ≥15 min, informed by prior work in *E. arcuata* as well as from our observations in *Portia*, in which REM-like episodes recur at intervals of ~20 to 30 min (1), to ensure that arousal testing targeted sustained inactivity occurring between REM-like episodes rather than transitional states. Response latency—time from stimulus onset to any visible reaction (arousal, startle, movement)—was recorded (see also Movie S2).

#### Sleep homeostasis.

To test for homeostatic regulation of sleep, we compared sleep patterns during baseline (phases 1 to 2) and rebound (phases 4 to 5) periods following sleep disruption in *Portia* [n = 17; 13 females (5 adults, 8 subadults), 4 males (4 subadults)]. Individuals were observed over a 5-phase protocol spanning 3 consecutive days: 1) baseline night, 2) baseline day, 3) sleep-disruption night, 4) rebound day, and 5) rebound night. Each phase lasted 12 h and was conducted under the same controlled environmental conditions described above.

Spiders were filmed individually in transparent enclosures (6 × 6 × 4 cm) and transferred into their filming boxes 2 h before lights-off to acclimate to the new environment. A moist cotton ball was provided for hydration. The sides of the enclosures were covered with opaque tape to prevent visual interactions between spiders.

After the 24-h baseline recording, sleep disruption was induced at the onset of the disruption night by introducing 25 live *D. melanogaster* (body length ≈ 3 mm) from our laboratory culture into each enclosure (see also Movie S3). A fresh cotton ball was added at the same time. The flies remained active and mobile throughout the night, moving freely within the enclosure.

Spiders were fed 1 to 2 d prior to the experiment, and no prey capture attempts or feeding events were observed during disruption nights. *P. fimbriata* specializes on spider prey, and although flies are accepted as prey, they do not typically elicit prolonged, fixated hunting behavior characteristic of spider predation. The presence of flies reliably suppressed REM-like bouts and disrupted prolonged quiescence. Spiders adopted typical resting postures, but these were repeatedly interrupted by brief movements, including repositioning, startle responses, and occasional attempts to dislodge flies. While brief or shallow sleep episodes cannot be fully excluded, the repeated behavioral interruptions preclude prolonged consolidated sleep. After 12 h, flies were removed and spiders were filmed for another 24 h to assess rebound effects on subsequent sleep.

This naturalistic, low-impact stimulus reliably disrupted REM-like bouts, while spiders remained in typical resting postures, minimizing potential confounds from forced locomotion or stress-induced arousal. Because brief or shallow sleep episodes may still have occurred, we refer to this manipulation as sleep disruption rather than complete sleep deprivation.

Due to storage and recording-time constraints of DSLR cameras, the disruption night was not filmed continuously; however, pilot trials confirmed that introducing flies under these conditions reliably impeded REM-like bouts. Up to 8 spiders were filmed per night using 4 DSLR cameras simultaneously (2 spiders per camera). This technically demanding setup occasionally required repeating sessions due to intermittent power or recording interruptions. Ultimately, high-quality recordings suitable for analysis were obtained from 13 (10 females, 3 males; subadults (n = 10) and adults (n = 3) of the 17 spiders (24 recordings in total). To test whether sleep window duration changed across consecutive nights independently of sleep disruption, five additional control spiders [2 females, 2 males, 1 unsexed; adults (n = 2) subadults (n = 2), and juveniles (n = 1)] were recorded for three consecutive undisturbed nights under otherwise identical experimental conditions.

#### Video scoring and statistical analyses.

Videos for the sleep architecture and homeostasis experiments were manually scored in BORIS (Behavioral Observation Research Interactive Software; 58) according to predefined behavioral categories (Table 1). All statistical analyses were conducted in R v4.4.3 (59). Data processing and visualization in R were performed primarily using the *tidyverse* and *ggplot2* packages (60, 61), We used generalized linear mixed-effects models (GLMMs; *glmmTMB* package; Brooks et al. (62) to analyze activity and sleep parameters, specifying model families and link functions appropriate to each response variable (Gamma distribution with log link for duration measures; Poisson distribution for count data). Random effects for individual identity were included where repeated measurements per individual occurred. For the sleep homeostasis experiment, baseline (phases 1 to 2) and rebound (phases 4 to 5) values were averaged per individual to enable direct comparisons of pre- and postdisruption conditions. Total sleep window, REM-like bout count, total time spent in REM-like sleep, and individual REM-like bout durations were analyzed as response variables. For the arousal threshold experiment, behavioral state at stimulus onset (awake, quiescent, REM-like) was included as a fixed effect to test for state-dependent differences in response latency. Because each trial involved a different individual, no random effect for individual identity was included in this analysis. Only trials with a measurable response were included in the latency model; nonresponses were summarized descriptively. Sex effects on response latency were tested in a separate model. Pairwise post hoc comparisons were performed using *emmeans* (63) and adjusted for multiple testing using Bonferroni correction. Model assumptions and fit were assessed using *DHARMa* (64), including tests for uniformity, dispersion, outliers, and zero inflation for count models. Sleep architecture metrics were calculated from BORIS files, and ethograms and associated figures were generated in Python (v. 3.14.2; 65) using *pandas* (66)*, NumPy* (67)*, Matplotlib* (68), and *seaborn* (69). Circadian activity data were also processed and visualized in Python using *Polars*, *NumPy*, and *Matplotlib*.

### AI Disclosure Statement.

In preparation of this manuscript, the authors used AI tools to assist with the following: Code production, specifically supporting the analysis (sleep architecture and sleep homeostasis) and plotting of data (Deepseek, ChatGPT, Gemini). AI tools were also used for grammar/spell checking, and minor translation assistance (Grammarly, ChatGPT), however, not for text production or figure generation. The authors reviewed all AI-generated code, and take full responsibility for ensuring all content is accurate, valid, and original.

### Inclusion and Diversity Statement.

The authors greatly value equity, diversity, and inclusion (EDI) in science. This study was conducted under consideration of EDI best practice. The authors come from five different countries (Germany, the Netherlands, Australia, Italy, and the United States) representing different career stages (PhD candidate, PI, undergraduate, professor). One or more of the authors self-identifies as a member of the LGBTQ+ community. We actively worked to promote gender balance (of first author) in our reference list (70, 71).

### Acknowledgment of Country.

We acknowledge the Djabugay (Tjapukai) people, the Traditional Custodians of the Kuranda region in Queensland, Australia, where the spiders used in our study originate. We pay our respects to their Elders past and present, their sustainable management of the land since the Dreaming, and their ongoing connection to land, water, country, and nature.

## Supplementary Material

Appendix 01 (PDF)

Movie S1. Example video showing sleep-related and REM-like behavioural markers in *Portia fimbriata*.

Movie S2. Example video showing behavioural states and responses during the arousal threshold experiment in *Portia fimbriata*.

Movie S3. Example video showing the experimental sequence and stimulus presentation in the sleep homeostasis experiment.

## Data Availability

All data, code, supplemental material, and a README file are available from Zenodo public repository under the following link: https://doi.org/10.5281/zenodo.18501385 (72). All other data are included in the manuscript and/or supporting information.
