# Supplementary material for: Behavioral evidence for two-stage sleep in spiders
Source: Proc Natl Acad Sci U S A. 2026 Oct 26;123(45):e2618030123. doi: 10.1073/pnas.2618030123 (PMC13752372; doi:10.1073/pnas.2618030123)
Supplement: Supplementary file 1 [file pnas.2618030123.sapp.pdf]

PNAS

Supporting Information for
Behavioral evidence for two-stage sleep in spiders

Nadja Geiger, Maxim Q. Capelle, Phillip Rosentreter, Marie E. Herberstein, Armin Bahl, Massimo De Agrò, Barrett A. Klein, Daniela C. Rößler

Co-corresponding authors: Nadja Geiger, Daniela C. Rößler
Email: ngeiger@uni-bonn.de, daniela.c.roessler@gmail.com

This PDF file includes:

Legends for Movies S1 to S3
Legends for Datasets S1 to S4
Legends for Code S1 to S4
Supplemental Figures S1 and S2, and corresponding Legends

Other supporting materials for this manuscript include the following:

Supplemental Movies S1 to S3
Datasets S1 to S4
Code S1 to S4

Movie S1. Example video showing sleep-related and REM-like behavioural markers in *Portia fimbriata*.

Movie S2. Example video showing behavioural states and responses during the arousal threshold experiment in *Portia fimbriata*.

Movie S3. Example video showing the experimental sequence and stimulus presentation in the sleep homeostasis experiment.

Dataset S1. Circadian activity dataset. Tracking data from three experimental conditions: constant darkness, constant light, and a control condition.

Dataset S2. Sleep architecture dataset. Includes individual sleep metrics and summary statistics.

Dataset S3. Arousal threshold dataset. Master table containing behavioural response data.

Dataset S4. Sleep homeostasis dataset. Master table containing sleep baseline, disruption and rebound measurements.

Code S1. Python script used for analysis of circadian activity datasets, creating heat maps.

Code S2. Python script used for analysis of the sleep architecture dataset.

Code S3. R Markdown script used for analysis of the arousal threshold dataset.

Code S4. R Markdown script used for analysis of the sleep homeostasis dataset.

All supplemental material described above, including movies, datasets, and code are available in the Zenodo repository: https://doi.org/10.5281/zenodo.18501385.

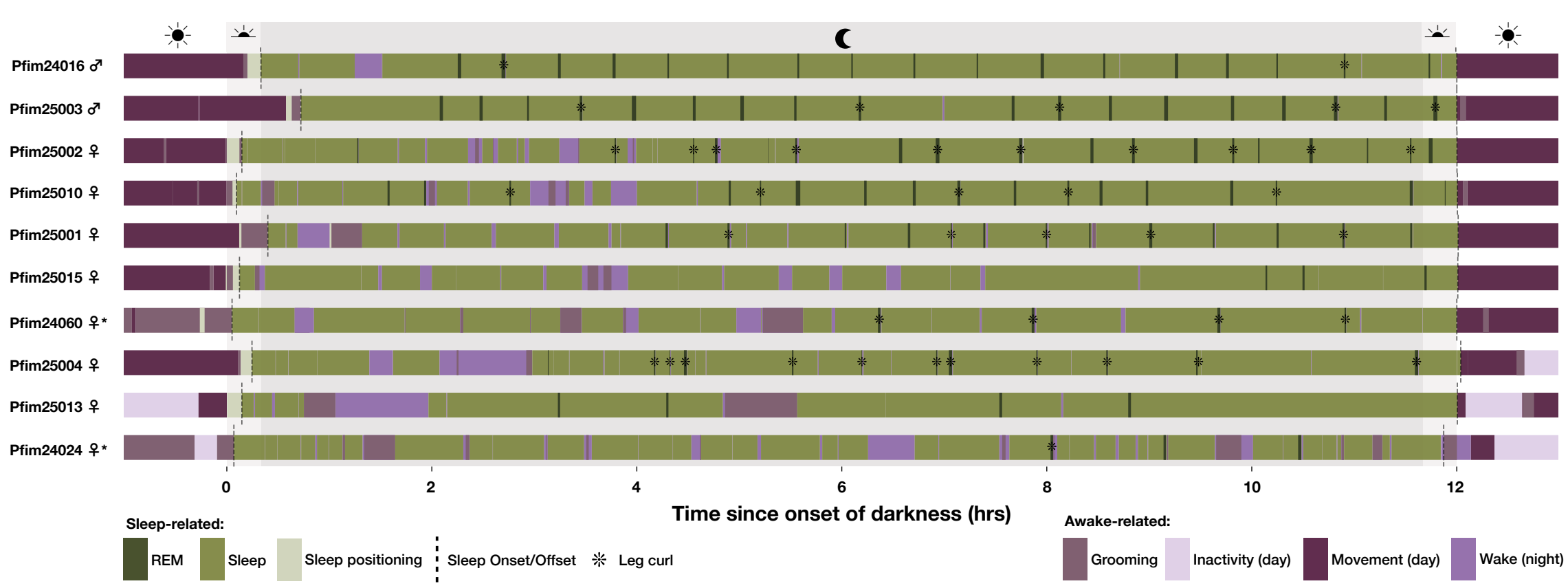

Supplemental Figure S1. Behavioral sequences for all subjects. Ethogram showing temporal organization of behavioral states and events in ten *Portia*. Distribution of distinct behavioral states (sleep, REM-like, leg curls, wake, grooming, positioning) over a 14-h period starting 1 h before and ending 13 h after the onset of darkness. Each spider is represented in its own horizontal row. Vertical dashed lines indicate sleep on- and offset; light transitions (lights-off at 0 h, lights-on at 12 h) are marked by background shading (dark phase = dark grey; twilight = light grey; light phase = no shading).

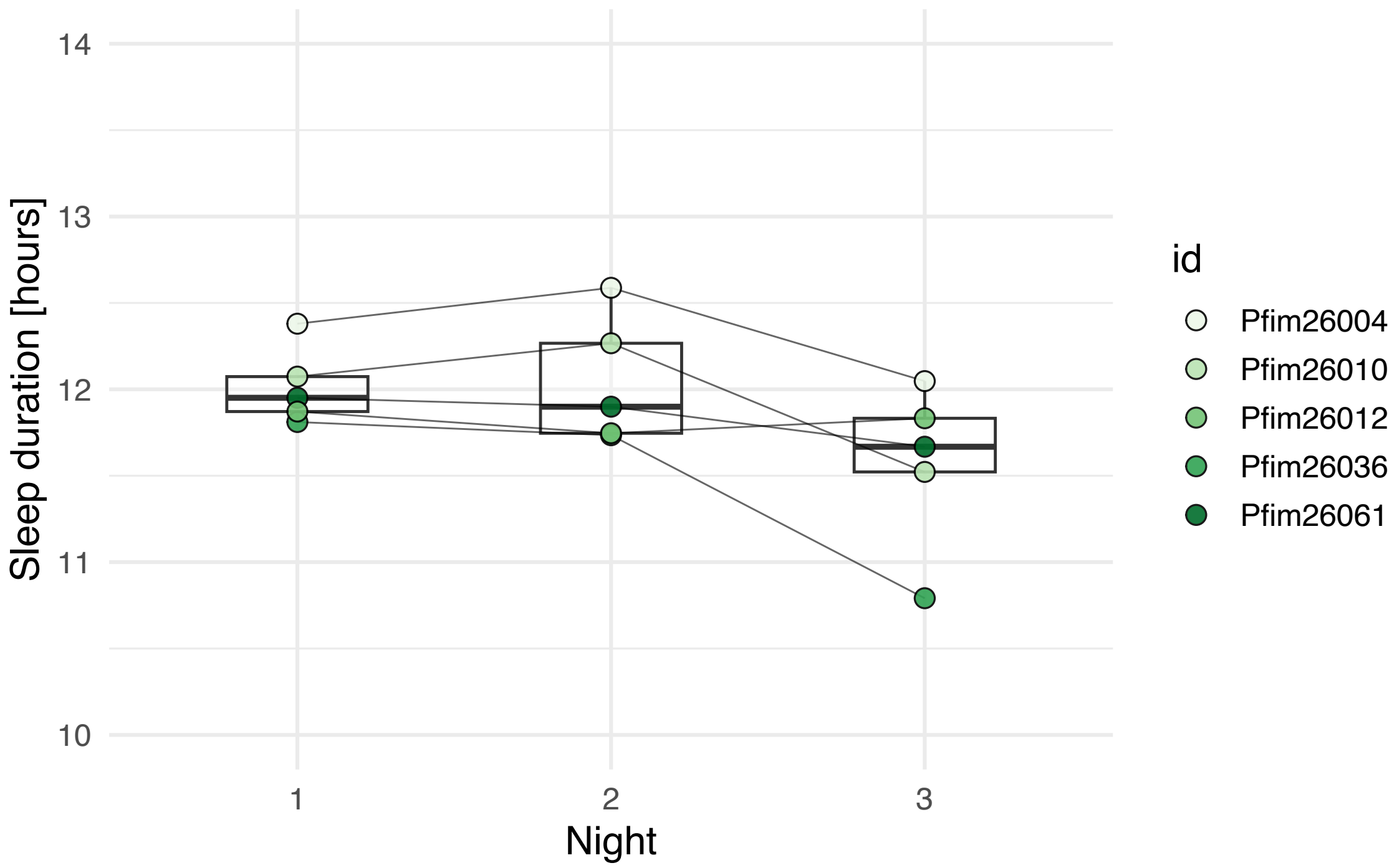

Supplemental Figure S2. Control experiment complementing sleep rebound data. The boxplots represent data collected for five additional spiders (*Portia fimbriata*), to test whether sleep duration changed across consecutive nights independently of sleep disruption. Spiders were recorded for three consecutive undisturbed nights (see x-axis) under otherwise identical experimental conditions (see sleep homeostasis experiment). Y-axis shows sleep duration in hours, and different shades of green represent 5 different spider individuals.
